# Supplementary material for: Exploiting the speckle-correlation scattering matrix for a compact reference-free holographic image sensor
Source: Nat Commun. 2016 Oct 31;7:13359. doi: 10.1038/ncomms13359 (PMC5095564; doi:10.1038/ncomms13359)
Supplement: Supplementary Information — Supplementary Figures 1&2, Supplementary Notes 1&2, Supplementary Methods and Supplementary References [file ncomms13359-s1.pdf]

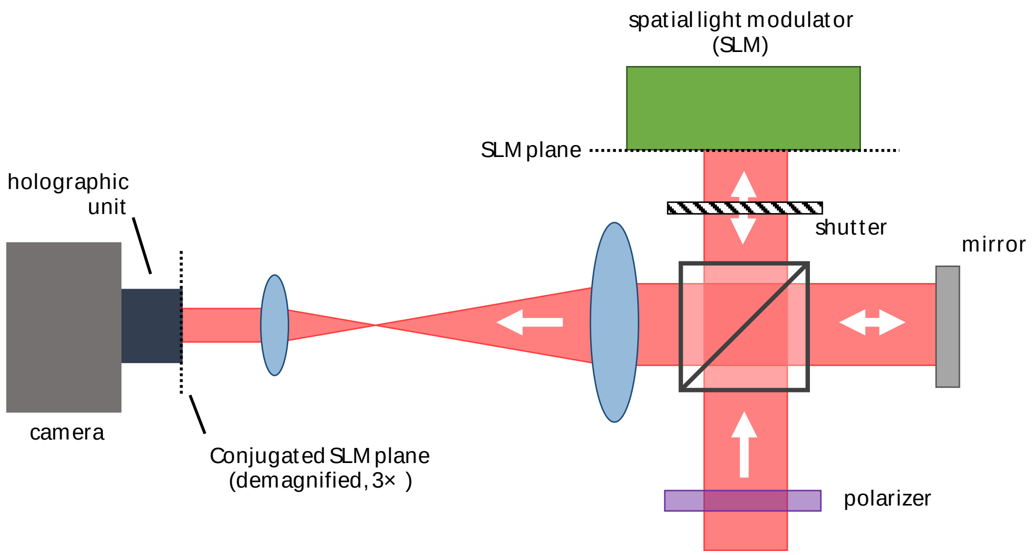

Supplementary Figure 1 | Experimental setup. Schematic of an optical setup for measuring a transmission matrix.

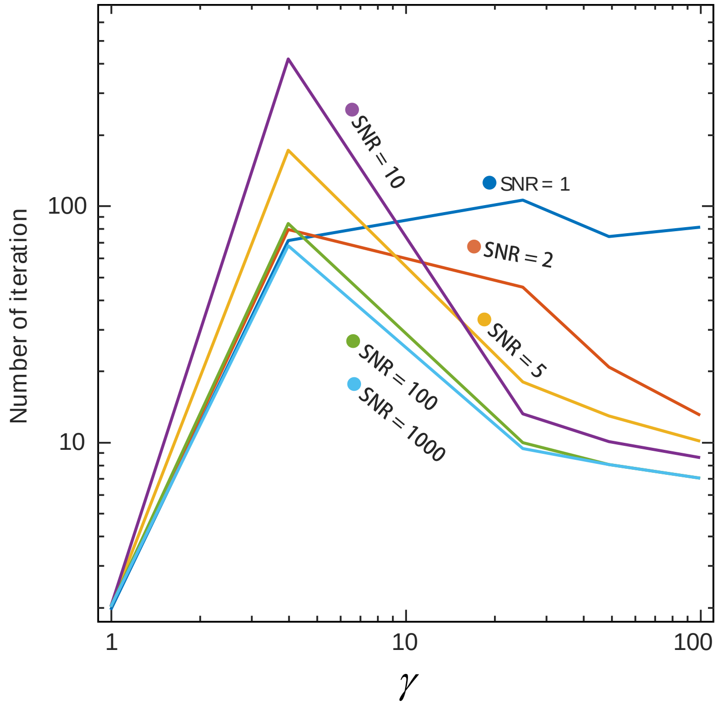

Supplementary Figure 2 | Mean number of iteration as a function of M to N ratio (γ) and SNR. The total number of iterations decreases as γ increases. However, because large γ requires a large transmission matrix for a given N, the computation time for each iteration would proportionally increase.

Supplementary Note 1: Required conditions for the speckle-correlation scattering matrix.

Since the principle of the present method relies on the mathematical moment relations of chaotic fields [Eq. (2)], it would work, regardless of physical properties of diffuser (i.e. thickness, degree of scattering, absorption) or the size of transmission matrix (TM) do not matter unless below two conditions are violated:

1. $t_1, t_2, \cdots, t_N$, and y are randomly diffused (complex Gaussian distributed).

   This is the reason why the 'diffusive' layer is necessarily required. In the case of an optical lens, for example, an output light field is well-ordered that does not satisfy the condition 1 as well as Eq. (2), unless the input field is already randomized. In the case of a diffuser, in contrary, condition 1 can be satisfied unless the input field is intentionally well shaped to make the output field be ordered.
2. Orthogonality between the column vectors of a TM, $\frac{1}{\sqrt{\Sigma_p \Sigma_q}} \left\langle t_p^* t_q \right\rangle_r = \delta_{pq}$.

   This condition is essential to retrieve a unique and exact solution, i.e. a correct optical field image of a sample. This assumption of orthogonality has been accepted for transport through a diffusive layer[1, 2, 3]

We note that regarding the condition 2, there exist (very few) correlated elements in a TM [4, 5, 6, 7, 8]. Nonetheless, even in such a situation, our method is still valid and can be successfully implemented by simply changing the basis of interest. Once the transmission matrix is calibrated, we can freely transform the basis. Then, the unwanted correlation elements can be filtered out by simply selecting the eigenvectors (or left-singular vectors) $u_1, u_2, \cdots, u_N$ (instead of $t_1, t_2, \cdots, t_N$) as the basis of interest, which ensures the condition 2. Since the eigenvectors of a diffusive layer are also randomly diffused in general, they also satisfy the condition 1. Thereby, we can reconstruct a hologram by constructing a Z matrix:

$$\mathbf{Z}_{pq} = \left\langle u_p^* u_q y^* y \right\rangle_r - \left\langle u_p^* u_q \right\rangle_r \left\langle y^* y \right\rangle_r , \tag{1}$$

where $\Sigma_{p,q} = \left\langle \left| u_{p,q} \right|^2 \right\rangle_r = 1$ in this case. Even if a TM is not a full-rank matrix (i.e. rank < N), it is still possible to impelement our method by filtering out the null space vectors. However, in such cases, the effective number of input basis would be decreased down to the rank of the transmission matrix.

Supplementary Note 2: Theoretical expectation of the intrinsic signal-to-noise ratio

Let us consider light transmission in a diffusive layer with a $M \times N$ TM matrix, **T**. Using singular value decomposition, we can rewrite the TM as $\mathbf{T = USV}^{\dagger}$, where **U** is a $M \times M$ matrix composed of orthonormal output basis vectors $\mathbf{u}_1, \mathbf{u}_2, \cdots, \mathbf{u}_M$; **V** is a $N \times N$ matrix composed of orthonormal input basis vectors $\mathbf{v}_1, \mathbf{v}_2, \cdots, \mathbf{v}_N$; and **S** is a $M \times N$ diagonal matrix, whose diagonal components are singular values whose elements satisfying $s_1 \geq s_2 \geq \cdots \geq s_N$.

Then, for an arbitrary input field $\mathbf{x} = \sum_{p=1}^{N} \beta_p \mathbf{v_p}$, transmitted light can be expressed as $\mathbf{y} = \mathbf{Tx} = \sum_{p=1}^{N} \beta_p \mathbf{u}_p$, which implies $\mathbf{u}_{p>N}^{\dagger} \mathbf{y} = 0$. Here, we define $\lambda_p = \mathbf{t}_p^{\dagger} \mathbf{y}$ and $\sigma_p = \mathbf{t}_p^{\dagger} \mathbf{y}^*$, which are the first and second term of Eq. (3), respectively. Since $\mathbf{t}_p = \mathbf{Tk}_p$, $\mathbf{t}_p$ can also be decomposed as $\mathbf{t}_p = \sum_{p=1}^{N} \chi_p \mathbf{u_p}$; therefore,

$$\begin{aligned} \lambda_p &= \left(\sum_{p=1}^{N} \chi_p^* \mathbf{u}_p{}^{\dagger} \right) \left(\sum_{p=1}^{N} \beta_p \mathbf{u}_p \right) = \sum_{p=1}^{N} \chi_p^* \beta_p \\ \sigma_p &= \left(\sum_{p=1}^{N} \chi_p^* \mathbf{u}_p{}^{\dagger} \right) \left(\sum_{p=1}^{N} \delta_p \mathbf{u}_p \right) = \sum_{p=1}^{N} \chi_p^* \delta_p \end{aligned}, \tag{2}$$

where $\mathbf{y}^* = \sum_{p=1}^{M} \delta_p \mathbf{u}_p$. The $\left|\lambda_p\right|^2$ and $\left|\sigma_p\right|^2$ are calculated as,

$$\begin{aligned} \left|\lambda_p\right|^2 &= \sum_{p,q=1}^{N} \chi_p^* \chi_q \beta_p^* \beta_q \approx \sum_{p=1}^{N} \left|\chi_p\right|^2 \left|\beta_p\right|^2 \\ \left|\sigma_p\right|^2 &= \sum_{p,q=1}^{N} \chi_p^* \chi_q \delta_p^* \delta_q \approx \sum_{p=1}^{N} \left|\chi_p\right|^2 \left|\delta_p\right|^2 \end{aligned}, \tag{3}$$

where coefficients χ_p, β_p, and δ_p are complex-Gaussian arbitrary values. The cross multiplication terms can be neglected for a sufficiently large *N*. Using the ambiguity of coefficients, we can simplify the $\left\langle \left|\lambda_p\right|^2 \right\rangle$ and $\left\langle \left|\sigma_p\right|^2 \right\rangle$ as,

$$\begin{aligned} \left\langle \left|\lambda_p\right|^2 \right\rangle &\approx \left\langle \sum_{p=1}^{N} \left|\chi_p\right|^2 \left|\beta_p\right|^2 \right\rangle = N \left\langle |\chi|^2 \right\rangle \left\langle |\beta|^2 \right\rangle \\ \left\langle \left|\sigma_p\right|^2 \right\rangle &\approx \left\langle \sum_{p=1}^{N} \left|\chi_p\right|^2 \left|\delta_p\right|^2 \right\rangle = N \left\langle |\chi|^2 \right\rangle \left\langle |\delta|^2 \right\rangle \end{aligned}. \tag{4}$$

From $\mathbf{t}_p^{\dagger} \mathbf{t}_p = \sum_{p=1}^{N} \left|\chi_p\right|^2$, $\left\langle |\chi|^2 \right\rangle = \frac{1}{N} \left\langle \mathbf{t}^{\dagger} \mathbf{t} \right\rangle$, and similarly, $\left\langle |\beta|^2 \right\rangle = \frac{1}{N} \left\langle \mathbf{y}^{\dagger} \mathbf{y} \right\rangle$. However, from $\mathbf{y}_p^{\dagger} \mathbf{y}_p = \sum_{p=1}^{M} \left|\delta_p\right|^2$, $\left\langle |\delta|^2 \right\rangle = \frac{1}{M} \left\langle \mathbf{y}^{\dagger} \mathbf{y} \right\rangle$. Note that δ spans whole *M*-dimensional output space,

while χ and β spans *N* subspace. Thereby, the intrinsic root-mean-squared signal-to-noise ratio can be calculated as follows:

$$\mathrm{SNR}_{\mathrm{intrinsic}} = \sqrt{\frac{\left\langle \left|\lambda_p \lambda_q^*\right|^2 \right\rangle_{p,q}}{\left\langle \left|\sigma_p \sigma_q^*\right|^2 \right\rangle_{p,q}}} = \frac{\left\langle \left|\lambda\right|^2 \right\rangle}{\left\langle \left|\sigma\right|^2 \right\rangle} = \frac{M}{N} = \gamma \tag{5}$$

In summary, as the ratio *M/N* increases, the intrinsic noise from the second term of Eq. (3) diminishes. Since *M* can be easily increased, for example, by enlarging the camera pixel number, *M/N* can be increased, and thus the signal-to-noise ratio of this method, which would be beneficial for practical applications.

[Supplementary Methods]

Detailed implementations of the proposed method.

In this section, the detailed experimental procedure is explained with a flow chart. The following diagram introduces the notation of our flowchart:

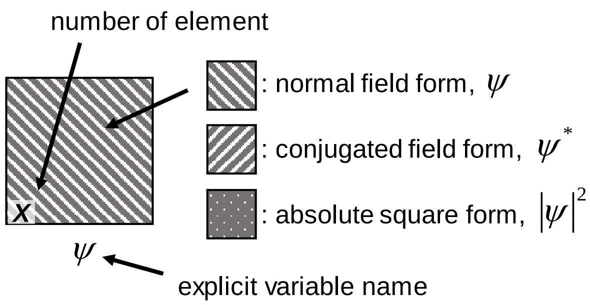

We employed the expressions in MATLAB® for the notation of element-wise multiplication (.*) and division (./), to represent pseudo algorithms for the procedure.

[Step 1: Transmission matrix calibration]

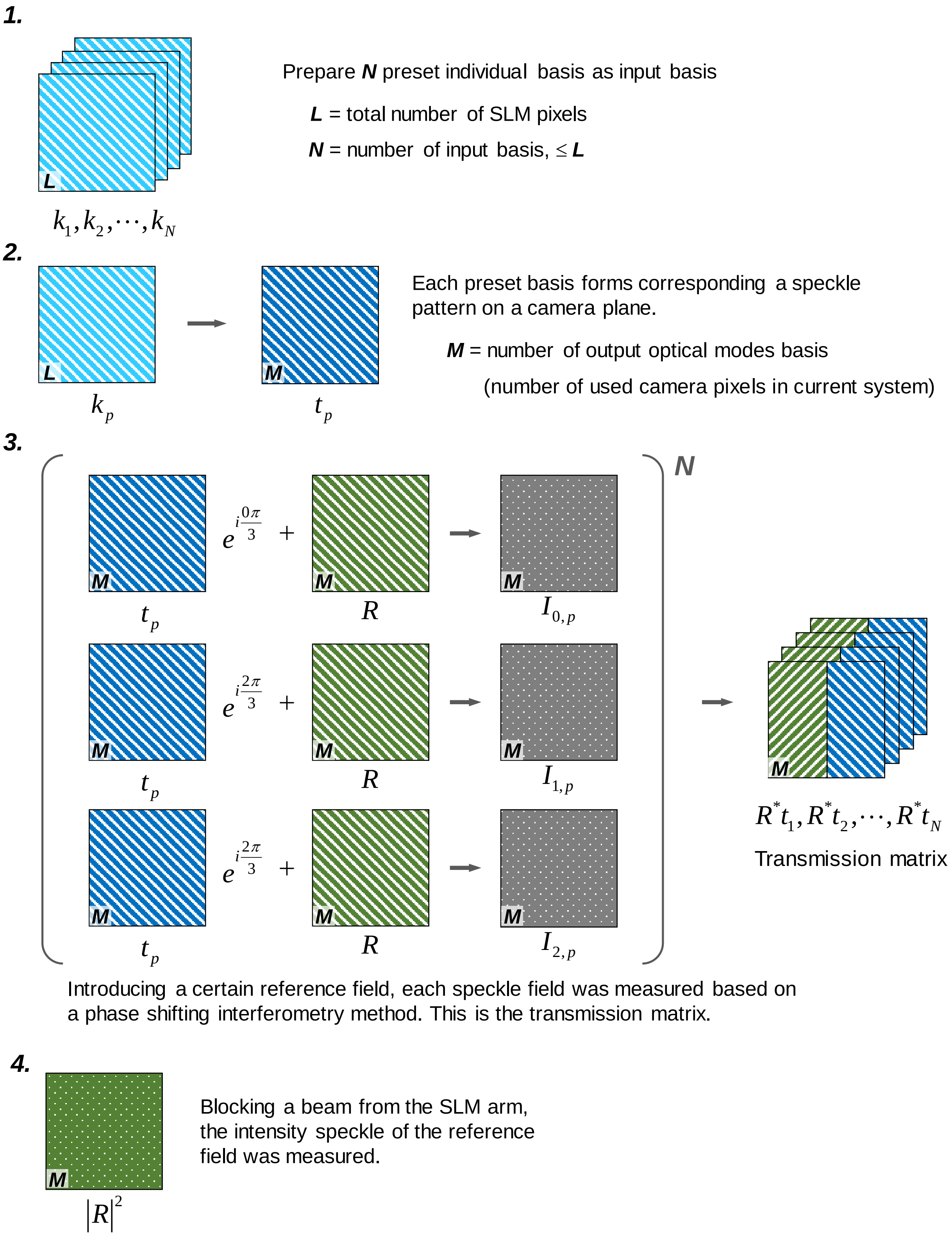

[Step 2: Field retrieval using the SSM method]

1.

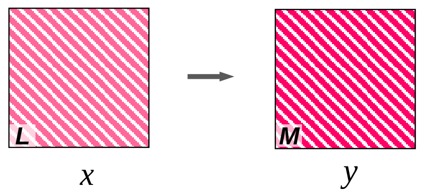

An unknown incident field forms a corresponding speckle pattern on the camera plane

2.

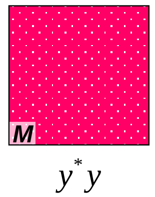

Measure the diffused intensity speckle of the incident field

3.

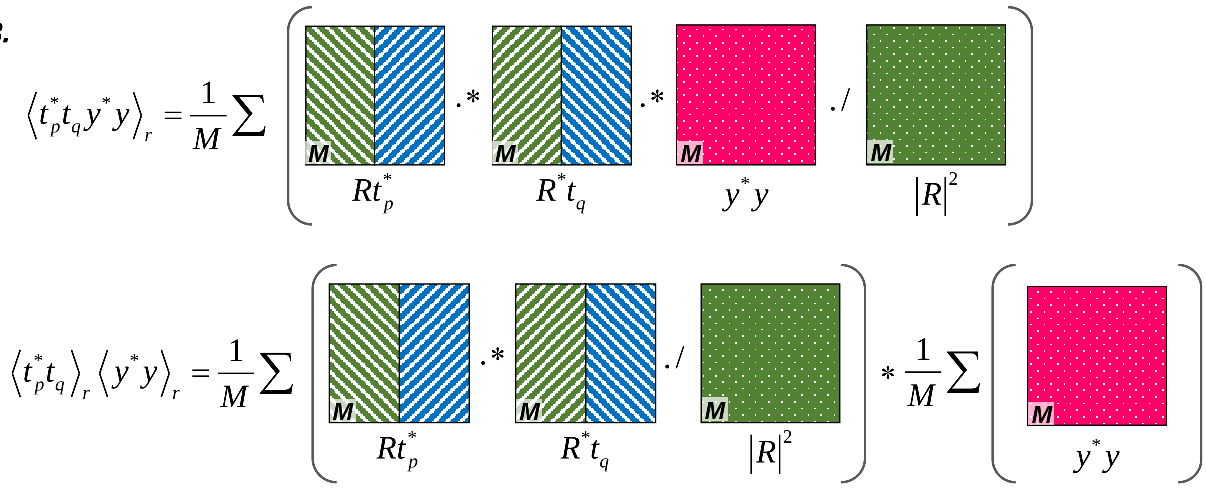

In order to construct the Z matrix, each term of Eq.(1) is calculated

4.

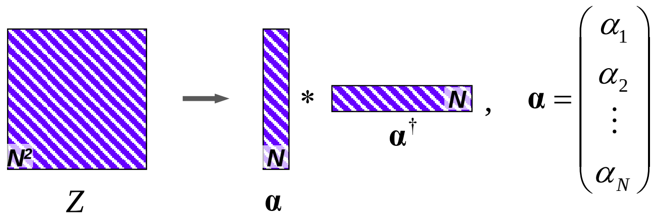

By decomposing the Z matrix, the eigenvector of the largest eigenvalue can be obtained

5.

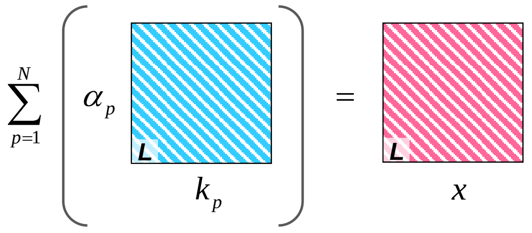

Using the coefficients, retrieve the incident field.

[(optional) Modified GS algorithm]

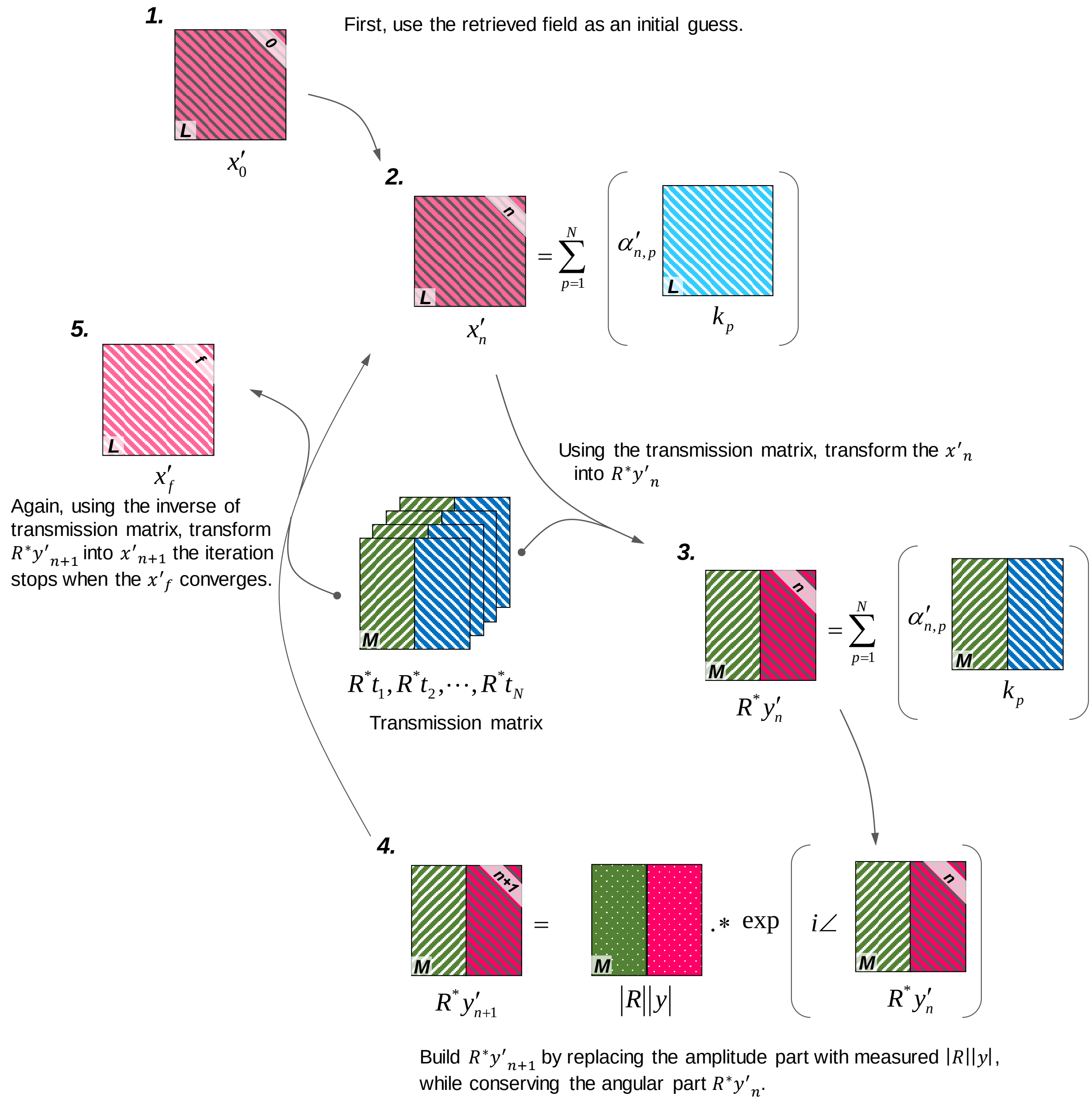

MATLAB® code for the numerical simulation

```
% Field retrieval using speckle-correlation scattering matrix (SSM), numerical simulation code.
% Written by KyeoReh Lee (Biomedical optics laboratory, KAIST, Korea), 2016-08-09
% for further information, please refer our group homepage: bmol.kaist.ac.kr


%% Initialization
gamma = 5*5;                        % M to N ratio. set this square number. only for #visualization. (related to line 29)
SNR = 100;                          % linear signal-to-noise ratio (SNR) to mimic practical noisy situations.

%% Incident field load
load('example_inputField.mat');     % load variable X, size(X) = [N, 1] complex double matrix.
N = numel(X);                       % set this square number. only for #visualization.
M = gamma*N;
X = X ./ norm(X);                   % normalize X for simplicity
X2d = reshape(X,[sqrt(N),sqrt(N)]); % 2D reshape. only for #visualization.

%% Transmission matrix (TM) and intensity speckle generation
disp('TM generate start!'); tic;
TM = raylrnd(sqrt(1/2/M),M,N).*exp(1i * random('unif',-pi,+pi-eps('double'),M,N));  % transmission matrix generation.
TM_prac = awgn(TM,SNR,'measured','linear');                                          % add noise upon preset SNR.
disp(['TM generate end! ... Elapsed time: ', num2str(toc),'s']);

Y = TM * X;                                                           % diffused field y generation.
Y_prac = awgn(Y,SNR,'measured','linear');                             % add noise upon preset SNR.
Y_prac2d = reshape(Y_prac,[sqrt(M),sqrt(M)]);                         % 2D reshape. #for visualization.
Iyy =  abs(Y_prac).^2;                                                % measured intensity speckle single shot.
Iyy2d_osampd = abs(fftshift(ifft2(ifftshift(...
   padarray(fftshift(fft2(ifftshift(Y_prac2d))),[2*sqrt(M),2*sqrt(M)]))))).^2;     % oversample Iyy. only for #visualization.

%% Field retrieval using SSM method
disp('initial guess start!'); tic;
sigma = mean(abs(TM_prac).^2,1);                                      % normalization factor, sigma (see Eq. (2)).
Zmatrix = (TM_prac'*(repmat(Iyy-mean(Iyy),[1,N]).*TM_prac))./(sigma'*sigma);        % Z matrix calculation.
[Xretv0,~] = eigs(Zmatrix,1);                                         % Field retrieval by selecting the eigenvector of the largest eigenvalue.
disp(['initial guess end! ... Elapsed time: ', num2str(toc),'s']);

Xcorr0 = X'*Xretv0/norm(X)/norm(Xretv0);                              % calculate correlation between X and Xretv0.
Xretv0 = Xretv0*exp(-1i*angle(Xcorr0));                               % global phase correction. only for #visualization.
Xretv02d = reshape(Xretv0,[sqrt(N),sqrt(N)]);                         % 2D reshape. only for #visualization.


%%  #Visualization
figure(1),
set(1,'Units', 'Normalized', 'OuterPosition', [0 0 1 1]);
subplot(243),imagesc(abs(X2d),[min(abs(X)),max(abs(X))]),axis image; title('incident amplitude'); colorbar
subplot(244),imagesc(angle(X2d)),axis image; title('incident wavefront'); colorbar
subplot(247),imagesc(abs(Xretv02d),[min(abs(X)),max(abs(X))]),axis image; title('retrieved amplitude'); colorbar
subplot(248),imagesc(angle(Xretv02d)),axis image; title(['retrieved wavefront, Corr. = ', num2str(abs(Xcorr0))]); colorbar
subplot(2,4,[1,2,5,6]), imagesc(Iyy2d_osampd,[0,max(Iyy2d_osampd(:))*0.5]), axis image; title('measured intensity speckle');
colormap(subplot(2,4,[1,2,5,6]),'gray');
pause(0.1);
```

```
%% (optional) Modified Gerchberg-Saxton algorithm
iterMax = 1000;                              % max iteration number to prevent infinite loop.
iterTor = 10^-5;                             % convergence criteria.

disp('inverse TM calculation start!'); tic;
[U,S,V]=svd(TM_prac,'econ');                 % economic svd for inverse TM calculation.
sv = diag(S);
TMinv_prac = V*(repmat(1./sv,[1,M]).*U');    % inverse TM calculation.
clearvars U S V
disp(['inverse TM calculation end! ... Elapsed time: ', num2str(toc),'s']);

disp('Modified Gerchberg-Saxton iteration start!'); tic;
Xcorr_iter = zeros(iterMax,1);               % archives correlation changes during iteration.
Xiter = Xretv0;                              % set retrieved field for the initial guess.
Xcorr_iter(1) = Xcorr0;

for itr = 1:1:iterMax

  Yiter = TM_prac * Xiter;                   % transform incident field into corresponding diffused field space.
  Yiter = sqrt(Iyy).*exp(1i*angle(Yiter));   % replace amplitude part with measured intensity.
  Xiter = TMinv_prac*Yiter;                  % return to the incident field space.
  Xcorr_iter(itr+1) = X'*Xiter/norm(Xiter);  % correlation calculation.

  if abs(Xcorr_iter(itr+1)) - abs(Xcorr_iter(itr)) < iterTor    % iteration ends when the criterion is satisfied.
    break;
  end
end
disp(['Modified Gerchberg-Saxton iteration end! ... Elapsed time: ', num2str(toc),'s']);

Xiter = Xiter*exp(-1i*angle(Xcorr_iter(itr)));                    % global phase correction. only for #visualization.
Xiter2d = reshape(Xiter,[sqrt(N),sqrt(N)])./ norm(Xiter);         % 2D reshape. only for #visualization.

%%  #Visualization
figure(2),
set(2,'Units', 'Normalized', 'OuterPosition', [0 0 1 1]);
subplot(241),imagesc(abs(X2d),[min(abs(X)),max(abs(X))]),axis image; title('incident amplitude'); colorbar
subplot(242),imagesc(angle(X2d)),axis image; title('incident wavefront'); colorbar
subplot(245),imagesc(abs(Xiter2d),[min(abs(X)),max(abs(X))]),axis image; title('retrieved amplitude'); colorbar
subplot(246),imagesc(angle(Xiter2d)),axis image; title(['retrieved wavefront, Corr. = ', num2str(abs(Xcorr_iter(itr+1)))]); colorbar
subplot(2,4,[3,4,7,8]), plot(abs(Xcorr_iter(1:itr+1))), title(num2str(itr))
pause(0.1);
```

Supplementary References

1. Popoff S, Lerosey G, Fink M, Boccara AC, Gigan S. Image transmission through an opaque material. *Nature Communications* **1**, 81 (2010).

2. Bertolotti J, van Putten EG, Blum C, Lagendijk A, Vos WL, Mosk AP. Non-invasive imaging through opaque scattering layers. *Nature* **491**, 232-234 (2012).

3. Katz O, Heidmann P, Fink M, Gigan S. Non-invasive single-shot imaging through scattering layers and around corners via speckle correlations. *Nature Photonics* **8**, 784-790 (2014).

4. Shi Z, Genack AZ. Transmission Eigenvalues and the Bare Conductance in the Crossover to Anderson Localization. *Physical Review Letters* **108**, 049301- (2012).

5. Vellekoop I, Mosk A. Universal optimal transmission of light through disordered materials. *Physical Review Letters* **101**, 120601 (2008).

6. Yu H, Park JH, Park Y. Measuring large optical reflection matrices of turbid media. *Optics Communications* **352**, 33-38 (2015).

7. Yu H, *et al.* Measuring large optical transmission matrices of disordered media. *Physical review letters* **111**, 153902 (2013).

8. Popoff SM, Lerosey G, Carminati R, Fink M, Boccara AC, Gigan S. Measuring the Transmission Matrix in Optics: An Approach to the Study and Control of Light Propagation in Disordered Media. *Physical Review Letters* **104**, 100601 (2010).
